# Matrix description of the complete topology of three-dimensional cells

**DOI:** 10.1038/srep25877

**Published:** 2016-05-10

**Authors:** Weihua Xue, Hao Wang, Guoquan Liu, Li Meng, Song Xiang, Guang Ma, Wenwen Li

**Affiliations:** 1 School of Materials Science and Engineering, University of Science and Technology Beijing, Beijing 100083, People’s Republic of China; 2 School of Materials Science and Engineering, Liaoning Technical University, Fuxin 123099, People’s Republic of China; 3 Collaborative Innovation Center of Steel Technology, University of Science and Technology Beijing, Beijing 100083, People’s Republic of China; 4 The State Key Laboratory for Advanced Metals and Materials, University of Science and Technology Beijing, Beijing 100083, People’s Republic of China; 5 Beijing R&D Department, East China Branch of Central Iron and Steel Research Institute, Beijing 100081, People’s Republic of China; 6 College of materials and metallurgy, Guizhou University, Guiyang 550025, People’s Republic of China; 7 Institute of Electrical and New Materials and Microelectronics, State Grid Smart Grid Research Institute, Beijing 102211, People’s Republic of China; 8 Department of Materials Engineering, The University of Tokyo, Tokyo 113-8656, Japan

## Abstract

A new, efficient method based on a series of matrices is introduced to completely describe the detailed topology of individual domains and their topology evolution in three-dimensional cellular structures. With this approach, we found a lot of new topological grain forms which are never reported before, i.e., there are total 8 and 32 topological forms for 7- and 8-faced grains respectively, other than the reported 7 and 27. This method is proved to be a practical tool to predict all possible grain forms efficiently. Moreover, a connectivity index of grain forms serves as a new convenient differentiator of different multicellular structures.

Metals and ceramics commonly consist of space-filling arrays of single-crystal grains separated by a network of grain boundaries, and foams (froths) are networks of gas-filled bubbles separated by liquid walls. Cellular structures or tessellations also occur in biological tissue, and in magnetic, ferroelectric and complex fluid contexts^1^.

Since the coarsening of cellular structures depends strongly on topology, the topological features of individual domains including grains, bubbles, cells, etc. in multicellular microstructures are subjects of longstanding interest which despite intensive studies is still not fully understood^1^,^2^,^3^,^4^,^5^,^6^,^7^,^8^,^9^,^10^. The most widespread notion relating to grain topology is the number of grain faces^3^,^4^,^5^,^6^,^7^,^8^. However, the number of faces of a grain is only a basic topological descriptor, it is clearly incomplete^9^,^10^. A more detailed topology of a grain should include the information of the number of faces, edges and vertices, as well as the adjacency relationships between each feature^2^. Of particular interest is how to describe such refined topology of grains completely and efficiently, with the ultimate object of throwing light on grain growth.

Nowadays, there are mainly two represent methods to describe the refined grain topology. Matzke^9^ introduced a more detailed topological description for bubbles in soap foams. Matzke characterized a large population of bubbles by recording the total number of faces and the number of edges of each face in each bubble. By extending this method, E.A. Lazar *et al*.^10^ introduced Weinberg vectors^11^ and **p** vectors^12^ to describe the grain topology and investigated the topological difference between Poisson-Voronoi and grain growth microstructures. Another method is the Schlegel diagram description of grains. Recently, B.R. Patterson *et al*.^13^ use Schlegel diagrams^14^ to describe the details of the topological flow of grains and generate some possible forms for grains with 4–9 faces.

The Schlegel diagrams of some grains, although not many in the system, are 2-connected but not 3-connected planar graphs^13^,^15^. The Weinberg vectors description is only suitable for grains with Schlegel diagrams being 3-connected planar graphs^10^, while not suitable for grains with Schlegel diagrams being 2-connected planar graphs. Moreover, it is difficult to generate the large number of possible grain forms efficiently by the Schlegel description of grain form evolution in grain growth, especially for grains with large face number. It is necessary to develop a general, efficient method to describe the refined grain topology which can overcome these disadvantages of the above two methods.

In this paper, based on spectral graph theory^16^, we develop a new, efficient method to completely classify the topology of three-dimensional grains and apply it in the study of the topological grain forms. It involves using a series of matrices to describe the grain topology, and using matrices operation to describe the grain form evolution in grain growth. With the aid of this method, we find that there are total 8 possible forms in 7-faced grain class, 32 forms in 8-faced grain class and 131 forms in 9-faced grain class respectively (See details in the Supplemental Material), which proved that a lot of grain forms are left out in the previous findings^13^.

## Methods

**Figure 1:**
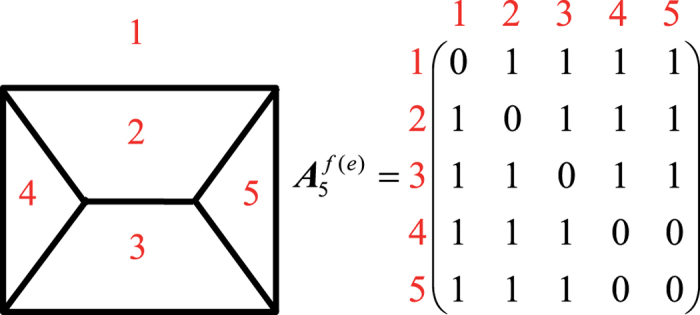
Schlegel diagram of a 5-faced grain and its face adjacency matrix.

We consider *n* to be the faces number of a grain, *p* the vertices number, and *q* the edges number. Euler’s Law relates the faces, edges, and vertices of a polyhedron as (*n* − *q* + *p* = 2)^17^. Under the restrictions of surface tension in three-dimensional cellular structures, three boundary interfaces meet at an edge and four edges meet at a vertex. Thus, for an individual grain, each edge has two vertices and each vertex radiates three edges, we can get (2*q* = 3*p*)^3^. We define the grain topological *face adjacency matrix* as the matrix***A***^*f*(*e*)^ = [*α*_*ij*_]_*n* × *n*_, in which *α*_*ij*_ = *k* if the face *i* and *j* have *k* joint edges, and *α*_*ij*_ = 0 if *i* and *j* have no joint edge or *i* = *j*. Figure 1 shows the Schlegel diagram of a 5-faced grain and its face adjacency matrix.

Topological properties like the number of grain faces, the number of edges per face, and the neighboring relations among different faces are involved in this matrix and can be calculated efficiently from matrix operation. The face adjacency matrix contains all the information of the correlations among grain faces, which can be used to completely describe the grain topology. We can also define the *vertex adjacency matrix* and *edge adjacency matrix*, however, they are not the complete topology description for grains whose Schlegel diagrams are not 3-connected graph.

The grains with same topological forms have isomorphic Schlegel diagram and permutation-similar ***A***^*f*(*e*)^. The spectra (i.e. the set of eigenvalues together with their multiplicities) of their ***A***^*f*(*e*)^ are identical. In spectral graph theory, the (adjacency) spectrum of a graph, which is the spectrum of its adjacency matrix, is one of its topological invariants^16^. Thus, the spectrum of ***A***^*f*(*e*)^ can be used to describe the grain topology.

The Laplacian matrix of a graph is defined as the degree matrix minus the adjacency matrix. For grain forms, the *face Laplacian matrix **L***^*f*(*e*)^ = diag(*d*(*f*_*i*_)) − ***A***^*f*(*e*)^, where diag(*d*(*f*_*i*_)) is a diagonal matrix in which the entries on main diagonal equal to the edge number of the grain faces. The second-smallest eigenvalue of the Laplacian matrix, *λ*_2_, is a very important parameter to describe the graph characteristics, which is a topological index and called algebraic connectivity^16^.

## Results and Discussions

### Topology characteristics of grain forms

Lazar^10^ presented eight most common grain topologies in grain growth and Poisson-Voronoi microstructure and observed that grain growth microstructure favor certain highly symmetric grain topologies relative to the Poisson-Voronoi microstructure. Table 1. listed their corresponding *λ*_2_ values. It can be seen that the most frequent grain forms in grain growth microstructure always has large *λ*_2_ values among all possible forms in a given face class. For 7-, 8-, 9- and 10-faced grains in grain growth microstructures, the most frequent grain topologies corresponds to 1^st^, 2^nd^,1^st^ and 1^st^ largest *λ*_2_ values in their total 8, 32, 131 and 723 forms, respectively. This reveals that some properties related to large *λ*_2_ are favored by grain growth, termed as algebraic connectivity preferred tendency (large *λ*_2_ preferred), besides the highly symmetric form preferred tendency observed in Lazar’s study^10^.

In spectral graph theory, large value of *λ*_2_ represents good connectivity, expansion and randomness properties of a graph^16^. Apparently, *λ*_2_ is influenced by not only the topological symmetry but also the edge distribution of grain faces, etc. For highly symmetric topological forms, if they have a narrow edge distribution of faces, i.e., the number of edges of each face approximates the average edges per face, the values of *λ*_2_ are large.

Actually, a narrow edge distribution of faces for a topological form is favorable in grain growth. Grain growth involves the form transition from higher face class to lower one by the disappearing of triangular faces. If a grain has more triangular faces, it increased the probability of face loss and transfer down the topological ladder to a disappearing tetrahedron to maintain grain growth^13^. And if a grain has no triangular face, it should employ edge-switch transitions to create triangular faces before decreasing to lower face classes. For a larger-edged face, the process of transferring down its edges ladder to ultimately a triangular face is complex relative to the smaller-edged faces. From the view of edge evolution, a larger-edged face of a grain is more stable than a smaller-edged faces. On the other hand, if one of grain faces increases its stability by increasing its edges, the edges of other faces are sure to decrease under the constraint of Euler’s equation. Therefore, the realistic way to increase the stability of a topological form is to modulate the edges of each face to approximate the average edges per grain face, which result in a narrow edge distribution of faces and a large *λ*_2_ value.

**Figure 2:**
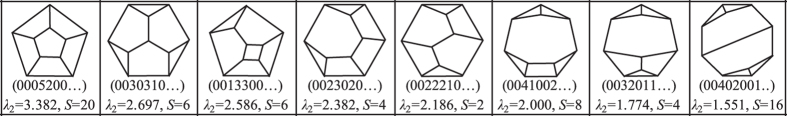
All eight 7-faced grain forms.

To make more clear of the difference between the algebraic connectivity and symmetry of topological forms, Fig. 2 lists all the topological forms of 7-faced grains together with the *λ*_2_ values and the order *S* of the symmetry forms. The *λ*_2_ value of each form decreased from left to right. The grain with **p** vector (0005200) has five 4-edged faces and two 5-edged faces, approximating to the average edges per face 4.2 for 7-faced grain class, has the largest *λ*_2_ value 3.382. The right-hand three forms have the smallest *λ*_2_ values, however, they have good symmetry, especially the most right hand one. These three forms are unstable and likely occur only as a transient state in the grain growth process, although they have high symmetry.

With the increase of triangular faces, the deviation degree from the average edges per face will increase, which resulted in low algebraic connectivity for grains. From the algebraic connectivity preferred tendency, these forms will have a less frequency in grain growth microstructures. This is in accord with Patterson’s result^13^ that the highest frequency forms have few or no triangular faces, while those with the most triangular faces are present the least.

The difference between the grain growth and Poisson-Voronoi microstructure can also be observed with respect to *λ*_2_ value in Table 1. The *λ*_2_ values of the most frequent grain topologies in grain growth microstructures are substantially larger than the corresponding ones in Poisson-Voronoi microstructures. This reveals that the availability of the matrix topology description can be used as another tool for distinguishing between fundamental characteristics of different cellular microstructures, besides the reported order of symmetry^10^ of grain topology in Weinberg-vector method.

### Grain form evolution in grain growth

An important application of the matrix topology description is the highly efficient analysis of the grain topological form evolution during the grain growth process. The fundamental topological events^13^ occurring in three-dimensional grain growth include: Type I – loss of a grain face through disappearance of a neighboring tetrahedron, Type II– grain encounter between grains not formerly touching and Type III – separation of grains sharing a face.

Steele^18^ used Schlegel diagrams and the topological events to study the collapsing polycrystal grains, illustrating the possible topological forms of grains by means of downward and horizontal transitions from 8 to 4 faces. Patterson^13^ expands Steele’s original analysis to include other possible grain forms resulting from uphill and horizontal transitions. However, it is difficult to work out all possible grain forms only by hands with the help of Schlegel diagrams and the topological events. That is part of the reasons why the above studies on topological forms are incomplete and terminated at 8-faced grains.

**Figure 3:**
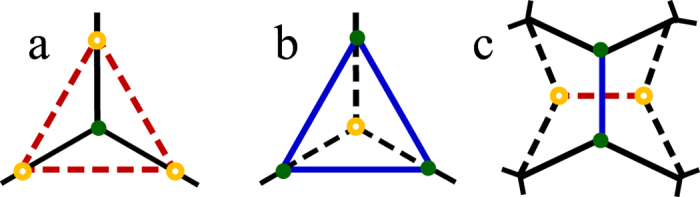
Changes of the vertices, edges and faces in Schlegel diagram during grain form evolution. (**a**) Losing a triangular face, (**b**) Gaining a triangular face and (**c**) Rearrangement of faces. The solid lines and dots denote the edges and vertices after evolution, respectively. The dashed lines and circles denote the original ones.

As the grain form evolves based on the topology events, the grain topological matrices will transform at the same time. We can use matrix operation to analyze the possible grain forms in grain growth efficiently. Through the fundamental topological events, a grain topological form would experience 3 possible changes: (1) losing a triangular face, (2) gaining a triangular face and (3) rearrangement of faces^13^. This can be reflected onto grain’s Schlegel diagram (See Fig. 3). Now we give the matrix description of the change (1) losing a triangular face as an example.

For convenience, three incidence matrices are constructed depending on the vertices, edges and faces of grain forms. (a) We define the grain topological *face*-*vertex incidence matrix* as ***M***^*f*−*v*^ = [*m*_*ij*_]_*n*×*p*_, in which *m*_*ij*_ = 1 when the vertex *j* is in the face *i*, and *m*_*ij*_ = 0 otherwise. (b) We define the grain topological *vertex*-*edge incidence matrix* as ***M***^*v*−*e*^ = [*μ*_*ij*_]_*p*×*q*_, in which *μ*_*ij*_ = 1 when the vertex *i* is in the edge *j*, and *μ*_*ij*_ = 0 otherwise. (c) We define the grain topological *face*-*edge incidence matrix* as ***M***^*f*−*e*^ = [*w*_*ij*_]_*n*×*q*_, in which *w*_*ij*_ = 1 when the edge *j* is in the face *i*, and *w*_*ij*_ = 0 otherwise. These three incidence matrices have correlations as follows,


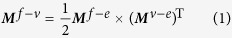


The generalized elementary matrices are used in the matrix operation, which include row-deleting matrix (***E***^*i*^, deleting row *i* of identity matrix, ***I***) and row-inserting matrix (***U***^*i*^, inserting a all 0’s row between row (*i*–1) and row *i* of ***I***), besides the elementary matrices as row-switching matrix (or permutation matrix, ***P***^*i*,*j*^), row-multiplication matrix (***D***^*i*(*c*)^, *c*≠0) and row-addition matrix (***T***^*i, j*(*k*)^). It should be noted that *c* could equal 0 in the generalized matrix ***D***^*i*(*c*)^.

For the change of (1) losing a triangular face, we consider that the *m*th face of an *n*-faced grain is a triangular face. Losing this face corresponds to the operation of deleting row *m* and the three columns with value “1” in ***M***_*n*_^*f*−*e*^. It is convenient to use the generalized elementary matrices to do matrix operation. That is


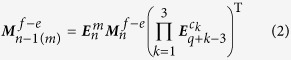


where 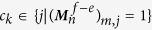, *c*_1_ < *c*_2_ < *c*_3_. Besides ***M***^*f*−*e*^,***M***^*f*−*v*^ and ***M***^*v*−*e*^ can also be obtained by matrix operation. The detailed derivations or explanations of all equations related to the above three topological changes can be seen in the Supplemental Material.

**Figure 4:**
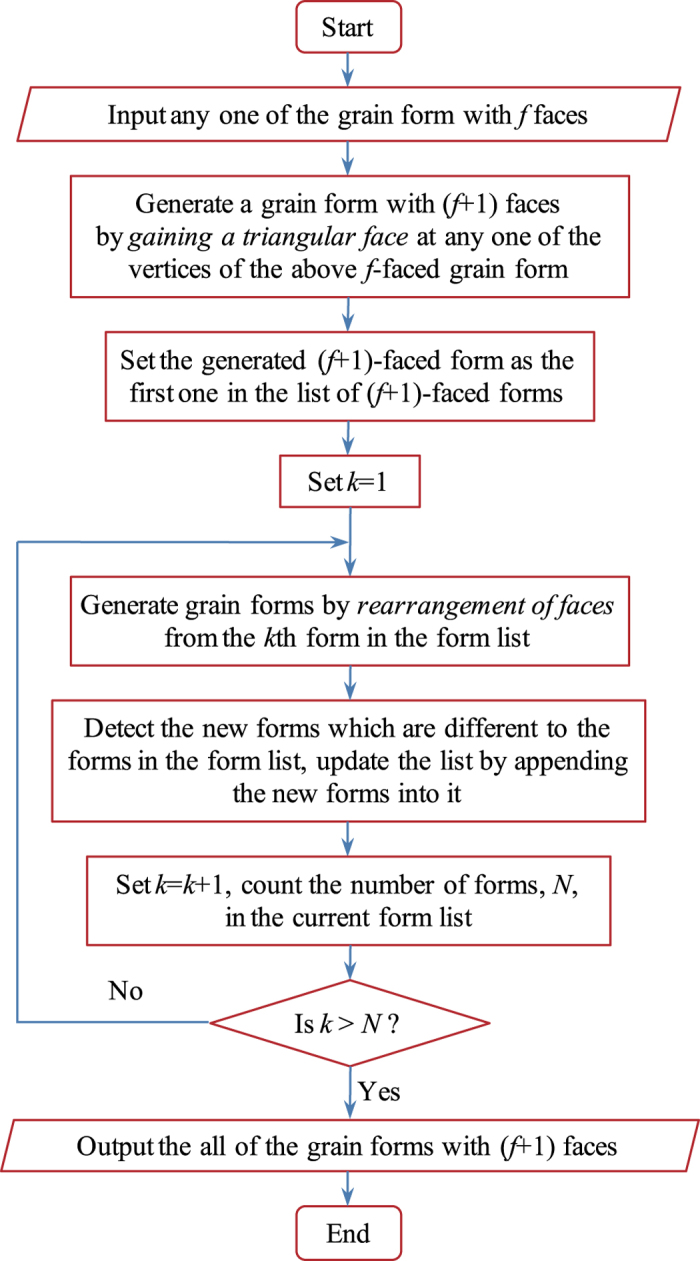
Flow chart of the generation of all the possible grain forms.

The most realistic means for determining possible grain forms is through the processes causing face-gain or loss in normal grain growth, i.e. encounter, separation and horizontal edge switch transitions within the same face class^13^. We begin with the lone tetrahedron in face class 4 and determine the possible higher forms gradually by uphill and horizontal topology transformations. Firstly, we place a triangular face at any one of the vertices of a grain form in lower face class to get a new grain form in higher face class. Secondly, we do the edge-switch transitions for this form and some other new forms would be created. Thirdly, we do edge-switch transitions again for all forms created in the previous step to find more new forms. The third step was repeated until there are no new forms created. Figure 4 shows the flow chart of the process of the generation of all the possible grain forms. The spectrum of ***A***^*f*(*e*)^ is used to distinguish whether a created form is a new one. Although having the same spectrum is only the necessary condition for two graphs to be isomorphic (i.e., the two forms are the same), it is a practice criterion because there have been found very few examples of non-isomorphic cubic graphs that have the same spectrum^19^.

**Figure 5:**
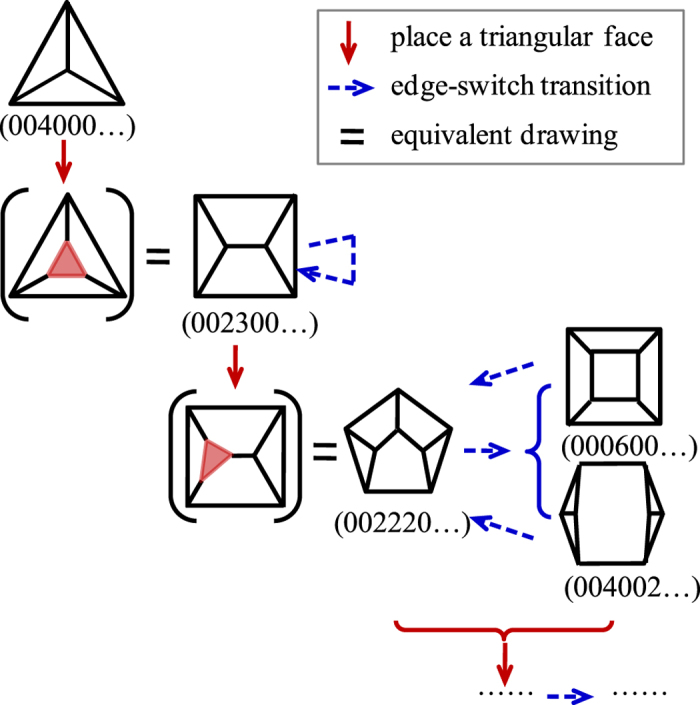
Sketch of the generation of 5- and 6-faced forms from the beginning 4-faced forms.

This method is able to generate all the possible grain forms, in that any one of *f*-faced forms can be transformed to all the other forms with *f* faces by the horizontal edge switch transitions, i.e., rearrangement of faces. The detailed proof is presented in the Supplemental Material and the path of the transition of any two 7-faced grain forms is also included as an example. Figure 5 shows the sketch of the generation of 5- and 6-faced forms from the beginning 4-faced forms.

The matrix description of grain form evolution in grain growth was implemented by developing a program written in Scilab 5.5.1 (free and open source software for numerical computation, Scilab Enterprises, available from: http://www.scilab.org). Table 2 lists the number of topological forms calculated by this program with a 4 × 3.40 GHz CPU, 16 G RAM computer in an acceptable period of time (e.g., 5 hours for 13-faced grains). For comparison, some similar results are also included. The enumeration of 3-regular simple (3-connected) and band-faced (2-connected but not 3-connected) planar graphs using a Monte Carlo algorithm is done by Keller^15^. The number of our calculated grain forms with 3-connected Schlegel diagrams in this work is the same as Keller’s for grains with 13 and fewer faces, but more larger than Keller’s results for grains with 14 faces. Especially, the number of our calculated band-faced planar graphs is larger than Keller’s results for grains with 8 and larger faces. This result indicates that many grain forms are not found by Monte Carlo method of which the reason is still clear. The generated simple graphs by *plantri* software are a little more than our and Keller’s results for grains with 13 and 14 faces, which is possibly because of the error in judging two same grain forms in different methods and the exact reason still awaits further analysis. The *plantri* software cannot generate band-faced planar graphs which limits its use in the grain growth studies.

**Figure 6:**
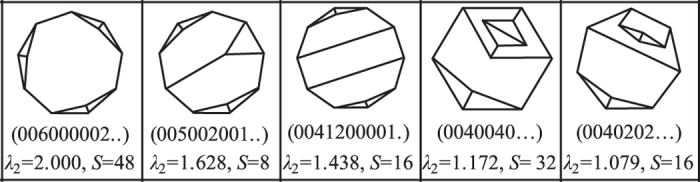
Five new forms found in this work beyond the known 27 forms for 8-faced grains as reported^13^.

We plot all the Schlegel diagrams for 8- and 9-faced grains in the Supplementary Material. Figure 6 lists 5 new forms found in this work beyond the known 27 forms for 8-faced grains as reported^13^. They all belong to band-faced topological grain forms and are observed to have characteristic pairs of contiguous three-edged faces. These three-edged face pairs are supposed to be unstable and decompose rapidly^13^ in grain growth process, resulting in relative low frequency of these grain forms in microstructures. It can also be seen that these forms in Fig. 5 have very high symmetry but wide edge distribution of grain faces. This further indicates that high symmetry is possibly a necessary condition for high frequency of occurrence of a certain grain form but not a sufficient condition. Those forms that occur more frequently than others normally have the characteristics of high symmetry plus narrow edge distribution of grain faces, which can be described by the new topology index found in this work-algebraic connectivity.

## Conclusion

In conclusion, we have introduced an efficient method to completely describe grain topologies. The application of this method to the grain form analysis has shown that the grain growth microstructure favors topological forms with large algebraic connectivity relative to the Poisson-Voronoi microstructure. This suggests that not only highly symmetric topological forms, but also forms with narrow edge distribution of faces are preferred in grain growth microstructure. The algebraic connectivity of grain forms has been proved to be a new convenient differentiator of different multicellular structures. Further, the matrix description of grain topology can serves as a new practical tool to predict the possible grain forms in three-dimensional grain growth efficiently.

## Additional Information

**How to cite this article**: Xue, W. *et al*. Matrix description of the complete topology of three-dimensional cells. *Sci. Rep.* **6**, 25877; doi: 10.1038/srep25877 (2016).

## Supplementary Material

Supplementary Information

## Figures and Tables

**Table 1:** The eight most common grain topologies in a descending order in the Poisson-Voronoi and grain growth microstructures^10^.

order	Poisson-Voronoi				Grain growth			
	p vector	*n*	λ_2_	ranking	p vector	*n*	λ_2_	ranking
1	(0013320)	9	2.350	**9**/131	(000440)	8	2.764	**2**/32
2	(0013310)	8	2.411	**5**/32	(000360)	9	3.000	**1**/131
3	(0004420)	10	2.463	**3**/723	(000441)	9	2.438	**4**/131
4	(0013411)	10	2.224	**18**/723	(000442)	10	2.463	**3**/723
5	(0004410)	9	2.438	**4**/131	(000520)	7	3.382	**1**/8
6	(0005220)	9	2.422	**6**/131	(000361)	10	2.786	**1**/723
7	(0014221)	10	2.180	**28**/723	(001330)	7	2.586	**3**/8
8	(0012520)	10	2.138	**35**/723	(001332)	9	2.350	**9**/131

Their **p** vectors, number of faces, algebraic connectivity λ_2_, and the ranking of λ_2_ in total forms of each face class are included.

**Table 2:** Summary of the grain forms generated using our matrix description method, Monte Carlo method^15^ and *plantri.*^15^

Face number	Matrix description method	Keller’s Monte Carlo				*plantri*
	Total forms	Simple	Band-faced	Simple	Band-faced	Simple
4	1	1	0	1	0	1
5	1	1	0	1	0	1
6	3	2	1	2	1	2
7	8	5	3	5	3	5
8	32	14	18	14	15	14
9	131	50	81	50	64	50
10	723	233	490	233	352	233
11	4345	1249	3096	1249	2096	1249
12	29,404	7595	21,809	7595	14,011	7595
13	210,839	49,565	161,274	49,565	98,119	49,566
14	1,584,418	339,714	1,244,704	327,848	376,266	339,722
