# Supplementary material for: Matrix description of the complete topology of three-dimensional cells
Source: Sci Rep. 2016 May 10;6:25877. doi: 10.1038/srep25877 (PMC4861958; doi:10.1038/srep25877)
Supplement: Supplementary Information [file srep25877-s1.pdf]

Supplementary Information

Matrix description of the complete topology of three-dimensional cells

Weihua Xue, Hao Wang, Guoquan Liu, Li Meng, Song Xiang, Guang Ma, Wenwen Li

This Supplementary Information contains I. the definitions and the related characteristics of grain topological matrices, II. the derivation of the matrix description of grain topology evolution, III. the topological matrices for 5-faced and 6-faced grains, IV. Proofs for the viewpoint of “one of f-faced forms can be transformed to all of the other forms with *f* faces by the horizontal edge switch transitions” and V. Schlegel diagram, *p* vector, and algebraic connectivity (λ_2) of 8- and 9-faced grains.

I. The definitions and the related characteristics of grain topological matrices

In Part I, we give the definitions of the edge and vertex adjacency matrix and the correlations among the grain adjacency matrix and incidence matrix.

Besides the face adjacency matrix proposed in the paper, we can also define the *vertex adjacency matrix* and *edge adjacency matrix*. The grain topological *vertex adjacency matrix* is defined as the matrix $A^{v(e)} = [a_{ij}]_{p\times p}$, in which a_{ij}=1 if the vertex i and j are in a same edge, and a_{ij}=0 if the vertex i and j are not in a same edge or $i = j$. The grain topological *edge adjacency matrix* is defined as the matrix $A^{e(v)} = [a_{ij}]_{p\times p}$, in which a_{ij}=1 if the edge i and j have joint vertices, and a_{ij}=0 if i and j have no joint vertices or $i = j$.

There are some correlations among the three grain topological adjacency matrices. We look upon all the vertices of a grain as the vertex set $V(G)$ and all the edges as the edge set $E(G)$ of the graph G=$G(V, E)$. The Schlegel diagram of a grain is the planar embedding of the graph $G(V, E)$. The vertex adjacency matrix of the grain is the adjacency matrix of G. The face adjacency matrix is the adjacency matrix of the *dual graph* of G. And, the edge adjacency matrix is the adjacency matrix of the *line graph* of G. The dual of a graph corresponds to its planar embedding. This is the reason why the face adjacency matrix is the complete

description of the grain topology.

The correlations among the grain topological incidence matrices proposed in the paper are in the below:

$$\boldsymbol{M}^{f-v}=\frac{1}{2}\boldsymbol{M}^{f-e}\times(\boldsymbol{M}^{v-e})^{T} \tag{I-1}$$

$$\boldsymbol{M}_{i,j}^{f-e}=w_{i,j}=\begin{cases}1, & (\boldsymbol{M}^{f-e}\times\boldsymbol{M}^{v-e})_{i,j}=2\\ 0, & (\boldsymbol{M}^{f-e}\times\boldsymbol{M}^{v-e})_{i,j}\neq 2\end{cases} \tag{I-2}$$

$$\boldsymbol{M}_{i,j}^{v-e}=m_{i,j}=\begin{cases}1, & [(\boldsymbol{M}^{f-v})^{T}\times\boldsymbol{M}^{f-e}]_{i,j}=2\\ 0, & [(\boldsymbol{M}^{f-v})^{T}\times\boldsymbol{M}^{f-e}]_{i,j}\neq 2\end{cases} \tag{I-3}$$

where Eq.(I-3) is only applicable for the 3-connected graph G.

The correlations among the grain topological adjacency and incidence matrices are in the below:

$$\boldsymbol{M}^{v-e}\times(\boldsymbol{M}^{v-e})^{\mathrm{T}}=\boldsymbol{A}^{v(e)}+\mathrm{diag}(d(v_i)) \tag{I-4}$$

$$\boldsymbol{M}^{f-v}\times(\boldsymbol{M}^{f-v})^{\mathrm{T}}=2\boldsymbol{A}^{f(e)}+\mathrm{diag}(d(f_i)) \tag{I-5}$$

$$(\boldsymbol{M}^{v-e})^{\mathrm{T}}\times\boldsymbol{M}^{v-e}=\boldsymbol{A}^{e(v)}+\mathrm{diag}(d(e_i)) \tag{I-6}$$

where diag($d(v_i)$), diag($d(f_i)$) and diag($d(e_i)$) are the vertex, face and edge degree matrix respectively. They are the diagonal matrix in which the entries on main diagonal line are $d(v_i)$ (the number of edges with a joint vertex i), $d(f_i)$ (the number of edges of face i) and $d(e_i)$ (the number of vertices of edge i). For the grain forms, $d(v_i)=3$ and $d(e_i)=2$.

II. The derivation of the matrix description of grain topology evolution

In Part II, we give the definition of generalized elementary matrices, the detailed derivation of matrix description of grain form evolution.

1) Generalized elementary matrices

We use a kind of generalized elementary matrices to describe the transformation of topological matrices in grain growth. That generalized elementary matrices are the extension of elementary matrices. Their definitions are as follows,

(1) Row-switching matrix (Permutation matrix), which is denoted by $\boldsymbol{P}(i,j)$, or $\boldsymbol{P}^{i,j}$, $\boldsymbol{P}_n^{i,j}$, is obtained by swapping row i and row j of the (nth-order) identity matrix.

(2) Row-multiplication matrix, which is denoted by $\boldsymbol{D}(i(c))$, or $\boldsymbol{D}^{i(c)}$, $\boldsymbol{D}_n^{i(c)}$, is obtained by multiplying the row i with a real number c of the (nth-order) identity matrix.

(3) Row-addition matrix, which is denoted by $\boldsymbol{T}(i, j(k))$, or $\boldsymbol{T}^{i,j(k)}$, $\boldsymbol{T}_n^{i,j(k)}$, is obtained by multiplying the row j by a real number k and adding it to the row i of the (nth-order) identity matrix.

(4) Row-deleting matrix, which is denoted by $\boldsymbol{E}(i)$, or $\boldsymbol{E}^i$, $\boldsymbol{E}_n^i$, is obtained by deleting the row i of the (nth-order) identity matrix. It is a (n-1)-by-n matrix.

(5) Row-inserting matrix, which is denoted by $\boldsymbol{U}(i)$, or $\boldsymbol{U}^i$, $\boldsymbol{U}_n^i$, is obtained by inserting an all 0 row vector between row (i-1) and row i of the (nth-order) identity matrix. That is to say, the row i to n of identity matrix becomes the row (i+1) to (n+1) of row-inserting matrix, and the row i of row-inserting matrix is all 0 row vector. Row-inserting matrix is a (n+1)-by-n matrix.

$\boldsymbol{P}(i,j)$, $\boldsymbol{D}(i(c))(c\neq 0)$ and $\boldsymbol{T}(i, j(k))$ are the standard elementary matrices. Left multiplication (pre-multiplication) by an elementary matrix represents elementary row operations, while right multiplication (post-multiplication) represents elementary column operations. A matrix multiplied by $\boldsymbol{D}(i(0))$ on its left [right]

side represents row i [column] of the matrix becoming '0' row [column] vector. A matrix is multiplied by $\boldsymbol{E}(i)$ on its left means deleting its ith row. A matrix is multiplied by the transposition of $\boldsymbol{E}(i)$ on its right means deleting its ith column. A matrix is multiplied by $\boldsymbol{U}(i)$ on its left means inserting a '0' row vector at row i. And, a matrix is multiplied by the transposition of $\boldsymbol{U}(i)$ on its right means inserting a '0' column vector on column i.

2) Losing a triangular face

For an n-faced grain, if one of its triangular faces (set it is the mth face and denoted by f_m) is losing, the edges and vertices on it will lose too, while the three edges which is connect with the face (e_1, e_2, e_3 in Fig.II-1) will be prolonged and converged on a new vertex (v_4).

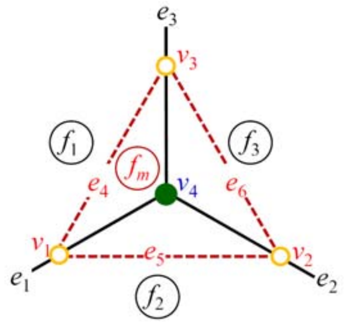

Fig.II-1 Sketch of losing a triangular face in Schlegel diagram of grain. The solid lines and dots denote the edges and vertices after transition, respectively. The dashed lines and circles denote the original ones.

The dimension of the face-edge incidence matrix of n-face grain ($\boldsymbol{M}_n^{f-e}$) is n-by-q (q is the number of grain edges and q =3(n-2)). Row m in $\boldsymbol{M}_n^{f-e}$ corresponds to the face f_{m} of the grain. The three columns with '1' values in row m correspond to the edges of face f_{m} (e_4, e_5, e_6 in Fig.II-1). Deleting row m and those three columns in $\boldsymbol{M}_n^{f-e}$ means the disappearing of the face f_m of the grain. Thus, the face-edge incidence matrix of the grain after the disappearance of a triangular face ($\boldsymbol{M}_{n-1(m)}^{f-e}$) can be produced and its dimension is (n-1)-by-(q-3). This procedure is shown in Fig.II-2. It could be written out by using generalized elementary matrices as below.

$$\boldsymbol{M}_{n-1(m)}^{f-e} = \boldsymbol{E}_n^m \boldsymbol{M}_n^{f-e} (\prod_{k=1}^{3} \boldsymbol{E}_{q+k-3}^{c_k})^{\mathrm{T}} \qquad \text{(II-1)}$$

where $c_k \in \{j \mid (\boldsymbol{M}_n^{f-e})_{m,j} = 1\}$, $c_1 < c_2 < c_3$.

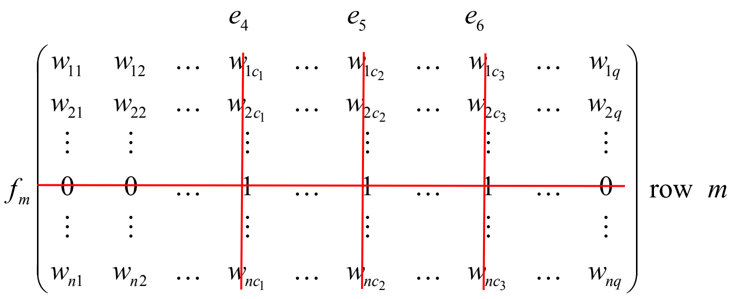

Fig.II-2 The form of $\boldsymbol{M}_n^{f-e}$ and the transition procedure of losing a triangular face. The red lines represent deleting the rows or columns of matrix.

For the vertex-edge incidence matrix of n-face grain ($\boldsymbol{M}_n^{v-e}$), its dimension is p-by-q (p is the number of grain vertices and p=2(n-2)). As shown in Fig.II-3, by deleting 3 rows (corresponds to 3 vertices) and 3 columns (corresponds to 3 edges), and then inserting a new row to generate a new vertex, the vertex-edge incidence matrix of the grain after the disappearance of a triangular face ($\boldsymbol{M}_{n-1(m)}^{v-e}$) can be obtained. In practice, it is convenient to get $\boldsymbol{M}_{n-1(m)}^{v-e}$ by adding any two rows to the third one and then deleting the two rows. The algorithm is,

$$\boldsymbol{M}_{n-1(m)}^{v-e} = \prod_{k=1}^{2} \boldsymbol{E}_{p+k-2}^{r_k} \prod_{k=1}^{2} \boldsymbol{T}_p^{r_3, r_k(1)} \boldsymbol{M}_n^{v-e} \left(\prod_{k=1}^{3} \boldsymbol{E}_{q+k-3}^{c_k}\right)^{\mathrm{T}} \qquad \text{(II-2)}$$

where $r_k \in \{j \mid (\boldsymbol{M}_n^{f-v})_{m,j} = 1\}$, $r_1 < r_2 < r_3$; and c_k is the same as Eq.(II-1).

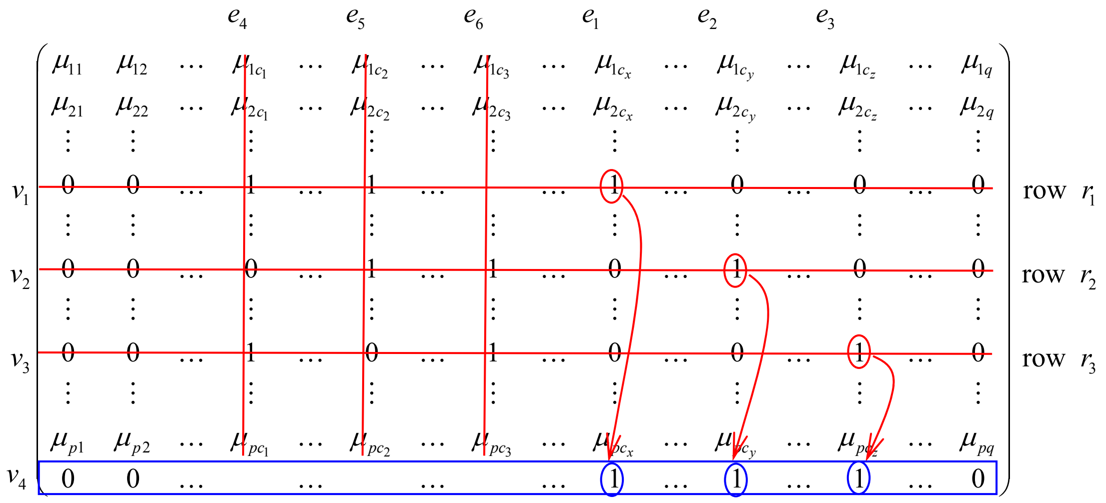

Fig.II-3 The form of $\boldsymbol{M}_n^{v-e}$ and the transition procedure of losing a triangular face. The red lines represent deleting the rows or columns, and the blue box represents inserting the rows of the matrix.

$\boldsymbol{M}_{n-1(m)}^{f-v}$ can be calculated out from $\boldsymbol{M}_{n-1(m)}^{f-e}$ and $\boldsymbol{M}_{n-1(m)}^{v-e}$ according to Eq.(2) in the article text or Eq.(I-1) in this Material.

3) Gaining a triangular face

When an *n*-faced grain gaining a triangular face, one of the vertices (set it is the *m*th vertex and denoted by v_m) will disappear, meanwhile, there will be three new vertices (v_1, v_2 and v_3) and three edges (e_4, e_5 and e_6) to emerge (see Fig.II-4).

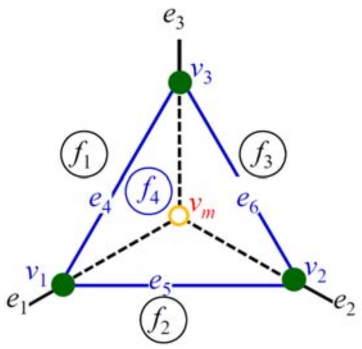

Fig.II-4 Sketch of gaining a triangular face in Schlegel diagram. The solid lines and dots denote the edges and vertices after transition, respectively. The dashed lines and circles denote the original ones.

Fig.II-5 shows the form of $\boldsymbol{M}_n^{f-e}$ and the transition procedure of gaining a triangular face. The algorithm is,

$$\boldsymbol{M}_{n+1(m)}^{f-e} = \boldsymbol{U}_n^{n+1}\boldsymbol{M}_n^{f-e}(\prod_{k=1}^{3}\boldsymbol{U}_{q-k+3}^{q-k+4})^{\mathrm{T}} + \prod_{k=1}^{n-3}\boldsymbol{U}_{n-k+1}^{r_k}\prod_{k=1}^{3}\boldsymbol{T}_4^{4,k(1)}\boldsymbol{U}_3^4(\prod_{k=1}^{q}\boldsymbol{U}_{q-k+3}^{1})^{\mathrm{T}} \qquad \text{(II-3)}$$

where, $r_k \in \{i \mid (\boldsymbol{M}_n^{f-v})_{i,m} = 0\}$, $r_1 > r_2 > \cdots > r_{n-3}$. The first part of the equation is to expand the original matrix to (*n*+1)-by-(*q*+3) dimension matrix using ‘0’ values, and the second part serves as filling new values to the matrix.

$$
\begin{array}{c}
 \\ \\ \\ f_1 \\ \\ f_2 \\ \\ f_3 \\ \\ \\ f_4
\end{array}
\begin{array}{c}
\begin{array}{cccc|ccc}
 & & & & e_4 & e_5 & e_6
\end{array}\\
\left(\begin{array}{cccc|ccc}
w_{11} & w_{12} & \cdots & w_{1q} & 0 & 0 & 0 \\
w_{21} & w_{22} & \cdots & w_{2q} & 0 & 0 & 0 \\
\vdots & \vdots & & \vdots & \vdots & \vdots & \vdots \\
w_{r_1 1} & w_{r_1 2} & \cdots & w_{r_1 q} & 1 & 0 & 0 \\
\vdots & \vdots & & \vdots & \vdots & \vdots & \vdots \\
w_{r_2 1} & w_{r_2 2} & \cdots & w_{r_2 q} & 0 & 1 & 0 \\
\vdots & \vdots & & \vdots & \vdots & \vdots & \vdots \\
w_{r_3 1} & w_{r_3 2} & \cdots & w_{r_3 q} & 0 & 0 & 1 \\
\vdots & \vdots & & \vdots & \vdots & \vdots & \vdots \\
w_{n1} & w_{n2} & \cdots & w_{nq} & 0 & 0 & 0 \\
\hline
0 & 0 & \dots & 0 & 1 & 1 & 1
\end{array}\right)
\end{array}
$$

Fig.II-5 The form of $\boldsymbol{M}_n^{f-e}$ and the transition procedure of gaining a triangular face. The blue box represents inserting the rows and columns of the matrix.

Fig.II-6 shows the form of $\boldsymbol{M}_n^{v-e}$ and the transition procedure of gaining a triangular face. The algorithm is,

$$
\begin{aligned}
\boldsymbol{M}_{n+1(m)}^{v-e} = & \prod_{k=1}^{3} \boldsymbol{U}_{p-k+2}^{p-k+3} \boldsymbol{E}_p^m \boldsymbol{M}_n^{v-e} \Big(\prod_{k=1}^{3} \boldsymbol{U}_{q-k+3}^{q-k+4}\Big)^{\mathrm{T}} + \prod_{k=1}^{p-1} \boldsymbol{U}_{p-k+2}^{1} \left(\prod_{k=1}^{q-3} \boldsymbol{U}_{q-k+3}^{c_k} \prod_{k=1}^{3} \boldsymbol{U}_{6-k}^{7-k} \right)^{\mathrm{T}} \\
& + \prod_{k=1}^{p-1} \boldsymbol{U}_{p-k+2}^{1} \prod_{k=1}^{q-3} \boldsymbol{E}_{k+3}^{c_{(q-k-2)}} (\boldsymbol{M}_n^{f-e})^{\mathrm{T}} \Big(\prod_{k=1}^{q} \boldsymbol{U}_{q-k+3}^{1} \prod_{k=1}^{n-3} \boldsymbol{E}_{k+3}^{r_{(n-k-2)}} \Big)^{\mathrm{T}}
\end{aligned} \tag{II-4}
$$

where, r_k is same as Eq.(II-3); $c_k \in \{ j \mid (\boldsymbol{M}_n^{v-e})_{m,j} = 0 \}$, $c_1 > c_2 > \cdots > c_{q-3}$. The first part of the equation is to delete the row m of $\boldsymbol{M}_n^{v-e}$ and to expand the matrix into (p+2)-by-(q+3) dimension using '0' values. The second part is to arrange incident edges of v_m in matrix and to expand the matrix into (p+2)-by-(q+3) dimension. The third part is to calculate $\boldsymbol{N}$ matrix using $\boldsymbol{M}_n^{f-e}$ and to expand the matrix into (p+2)-by-(q+3) dimension too.

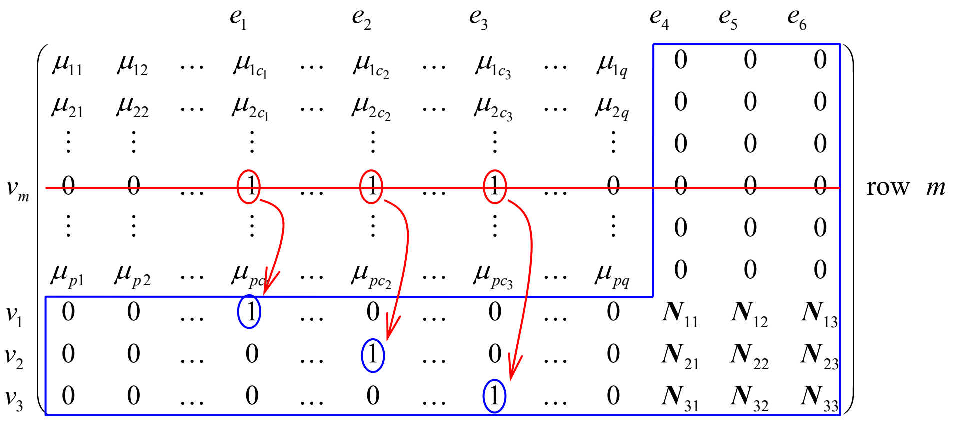

Fig.II-6 The form of $\boldsymbol{M}_n^{v-e}$ and the transition procedure of gaining a triangular face

4) Rearrangement of faces

When an n-faced grain rearrange the faces by doing edge-switching transition upon one of the edges (set it is the mth edge and denoted by e_m), the number of vertices, edges and faces will not change, but their arrangement would change. (see Fig.II-7).

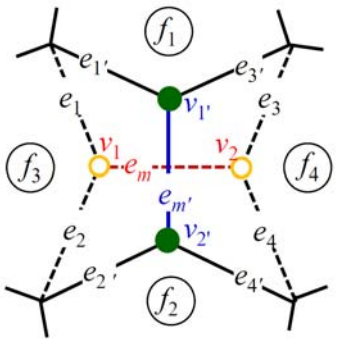

Fig.II-7 Sketch of rearrangement of faces in Schlegel diagram of grains

Fig.II-8 shows the form of $\boldsymbol{M}_n^{f-e}$ and the transition procedure of gaining a triangular face. The practical algorithm is,

$$\boldsymbol{M}_{n(m_s)}^{f-e} = \boldsymbol{M}_n^{f-e}\boldsymbol{D}_q^{m(0)} + \prod_{k=1}^{2}\boldsymbol{D}_n^{r_k(0)}\boldsymbol{M}_n^{f-v}\boldsymbol{M}_n^{v-e}(\boldsymbol{I}_q - \boldsymbol{D}_q^{m(0)}) \tag{II-5}$$

where, $r_k \in \{i\,|\,(\boldsymbol{M}_n^{f-e})_{i,m} = 1\}$. The former part of the equation is to make the column m into all '0' column. The latter part is to calculate the translated column m and to expand it into n-by-q dimension.

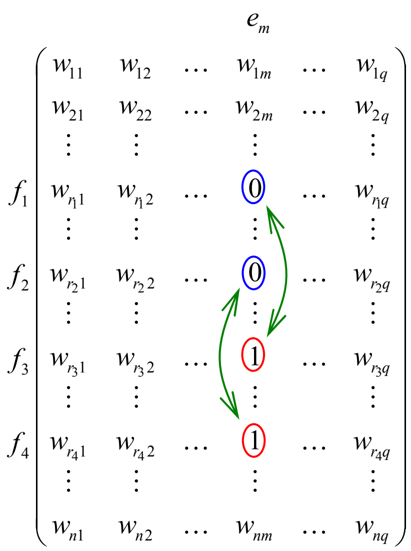

Fig.II-8 The form of $\boldsymbol{M}_n^{f-e}$ and the transition term. "0" values in blue circle become "1" and "1"values in red circle become "0".

Fig.II-9 shows the form of $\boldsymbol{M}_n^{v-e}$ and the transition procedure of gaining a triangular face. The algorithm is,

$$\boldsymbol{M}_{n(m_s)}^{v-e} = \prod_{k=1}^{2} \boldsymbol{D}_p^{c_k(0)} \boldsymbol{M}_n^{v-e} + (\boldsymbol{I}_p - \prod_{k=1}^{2} \boldsymbol{D}_p^{c_k(0)}) \boldsymbol{M}_n^{v-e} \boldsymbol{P}_q^{a,b} \tag{II-6}$$

where, $c_k \in \{i \mid (\boldsymbol{M}_n^{v-e})_{i,m} = 1\}$. The former part of the equation is to make the two columns which need to be exchanged into all '0' column. The latter part is to make the others into all '0' and to exchange the values in the rows which correspond to the two vertices in e_m. The number of the two exchanged columns a and b can be calculated from the below methods.

set $\boldsymbol{A}_s^{e(v)} = \prod_{k=1}^{q-4} \boldsymbol{D}_q^{s_k(0)} (\boldsymbol{M}_n^{v-e})^{\mathrm{T}} \boldsymbol{M}_n^{v-e} \prod_{k=1}^{q-4} \boldsymbol{D}_q^{s_k(0)}$, $\boldsymbol{A}_s^{e(f)} = \prod_{k=1}^{q-4} \boldsymbol{D}_q^{s_k(0)} (\boldsymbol{M}_n^{f-e})^{\mathrm{T}} \boldsymbol{M}_n^{f-e} \prod_{k=1}^{q-4} \boldsymbol{D}_q^{s_k(0)}$

where, $s_k \in \{i \mid ((\boldsymbol{M}_n^{v-e})^{\mathrm{T}} \boldsymbol{M}_n^{v-e})_{i,m} \neq 1\}$

then $a \in \{j \mid (\boldsymbol{A}_s^{e(v)})_{t_k, j} = 1\}$, $b \in \{j \mid (\boldsymbol{A}_s^{e(f)})_{t_k, j} = \max(\boldsymbol{A}_s^{e(f)})_{t_k}, j \neq t_k, j \neq a\}$

where, $t_k \in \{i \mid ((\boldsymbol{M}_n^{v-e})^{\mathrm{T}} \boldsymbol{M}_n^{v-e})_{i,m} = 1\}$, k is any one of 1, 2, 3 or 4.

$$
\begin{array}{c}
\\
\\
\\
v_1\\
\\
v_2\\
\\
\\
\end{array}
\begin{array}{c}
\begin{array}{cccccccccccccc}
 & & & e_1 & & e_2 & & e_3 & & e_4 & & & & \\
\end{array}\\
\begin{pmatrix}
\mu_{11} & \mu_{12} & \cdots & \mu_{1s_1} & \cdots & \mu_{1s_2} & \cdots & \mu_{1s_3} & \cdots & \mu_{1s_4} & \cdots & \mu_{1m} & \cdots & \mu_{1q} \\
\mu_{21} & \mu_{22} & \cdots & \mu_{2s_1} & \cdots & \mu_{2s_2} & \cdots & \mu_{2s_3} & \cdots & \mu_{2s_4} & \cdots & \mu_{2m} & \cdots & \mu_{2q} \\
\vdots & \vdots & & \vdots & & \vdots & & \vdots & & \vdots & & \vdots & & \vdots \\
0 & 0 & \cdots & 1 & \cdots & \textcircled{1} & \longleftrightarrow & \textcircled{0} & \cdots & 0 & \cdots & 1 & \cdots & 0 \\
\vdots & \vdots & & \vdots & & \vdots & & \vdots & & \vdots & & \vdots & & \vdots \\
0 & 0 & \cdots & 0 & \cdots & \textcircled{0} & \longleftrightarrow & \textcircled{1} & \cdots & 1 & \cdots & 1 & \cdots & 0 \\
\vdots & \vdots & & \vdots & & \vdots & & \vdots & & \vdots & & \vdots & & \vdots \\
\mu_{p1} & \mu_{p2} & \cdots & \mu_{ps_1} & \cdots & \mu_{ps_2} & \cdots & \mu_{ps_3} & \cdots & \mu_{ps_4} & \cdots & \mu_{pm} & \cdots & \mu_{pq}
\end{pmatrix}
\end{array}
\begin{array}{l}
\\
\\
\\
\text{row } c_1\\
\\
\text{row } c_2\\
\\
\\
\end{array}
$$

Fig.II-9 The form of $\boldsymbol{M}_n^{v-e}$ and the transition term (in red circle)

III. The topological matrices for 5-faced and 6-faced grains

1) The topological matrices for a 5-faced grain (00230…)

For convenience, the labeled Schlegel diagram of the 5-faced grain with p-vector (00230…) in Fig. 1 in the article text is re-plotted in here as Fig. III-1.

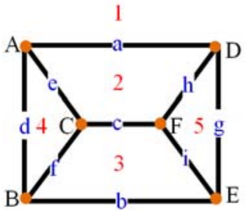

Fig. III-1 The vertices, edges and faces in the Schlegel diagram of a 5-faced grain. The faces are denoted by numbers, the vertices by capital letters, and the edges by lowercase letters.

Its face adjacency matrix is

$$\boldsymbol{A}_5^{f(e)} = \begin{array}{c} \\ 1 \\ 2 \\ 3 \\ 4 \\ 5 \end{array} \begin{array}{c} \begin{array}{ccccc} 1 & 2 & 3 & 4 & 5 \end{array} \\ \begin{pmatrix} 0 & 1 & 1 & 1 & 1 \\ 1 & 0 & 1 & 1 & 1 \\ 1 & 1 & 0 & 1 & 1 \\ 1 & 1 & 1 & 0 & 0 \\ 1 & 1 & 1 & 0 & 0 \end{pmatrix} \end{array}$$

Its face Laplacian matrix is

$$\boldsymbol{L}_5^{f(e)} = \begin{array}{c} \\ 1 \\ 2 \\ 3 \\ 4 \\ 5 \end{array} \begin{array}{c} \begin{array}{ccccc} 1 & 2 & 3 & 4 & 5 \end{array} \\ \begin{pmatrix} 4 & -1 & -1 & -1 & -1 \\ -1 & 4 & -1 & -1 & -1 \\ -1 & -1 & 4 & -1 & -1 \\ -1 & -1 & -1 & 3 & 0 \\ -1 & -1 & -1 & 0 & 3 \end{pmatrix} \end{array}$$

Its vertex adjacency matrix is

$$
\boldsymbol{A}_5^{v(e)} = \begin{array}{c} \\ \mathrm{A} \\ \mathrm{B} \\ \mathrm{C} \\ \mathrm{D} \\ \mathrm{E} \\ \mathrm{F} \end{array}
\begin{array}{c} \begin{array}{cccccc} \mathrm{A} & \mathrm{B} & \mathrm{C} & \mathrm{D} & \mathrm{E} & \mathrm{F} \end{array} \\
\begin{pmatrix} 0 & 1 & 1 & 1 & 0 & 0 \\ 1 & 0 & 1 & 0 & 1 & 0 \\ 1 & 1 & 0 & 0 & 0 & 1 \\ 1 & 0 & 0 & 0 & 1 & 1 \\ 0 & 1 & 0 & 1 & 0 & 1 \\ 0 & 0 & 1 & 1 & 1 & 0 \end{pmatrix} \end{array}
$$

Its edge adjacency matrix is

$$
\boldsymbol{A}_5^{e(v)} = \begin{array}{c} \\ \mathrm{a} \\ \mathrm{b} \\ \mathrm{c} \\ \mathrm{d} \\ \mathrm{e} \\ \mathrm{f} \\ \mathrm{g} \\ \mathrm{h} \\ \mathrm{i} \end{array}
\begin{array}{c} \begin{array}{ccccccccc} \mathrm{a} & \mathrm{b} & \mathrm{c} & \mathrm{d} & \mathrm{e} & \mathrm{f} & \mathrm{g} & \mathrm{h} & \mathrm{i} \end{array} \\
\begin{pmatrix} 0 & 0 & 0 & 1 & 1 & 0 & 1 & 1 & 0 \\ 0 & 0 & 0 & 1 & 0 & 1 & 1 & 0 & 1 \\ 0 & 0 & 0 & 0 & 1 & 1 & 0 & 1 & 1 \\ 1 & 1 & 0 & 0 & 1 & 1 & 0 & 0 & 0 \\ 1 & 0 & 1 & 1 & 0 & 1 & 0 & 0 & 0 \\ 0 & 1 & 1 & 1 & 1 & 0 & 0 & 0 & 0 \\ 1 & 1 & 0 & 0 & 0 & 0 & 0 & 1 & 1 \\ 1 & 0 & 1 & 0 & 0 & 0 & 1 & 0 & 1 \\ 0 & 1 & 1 & 0 & 0 & 0 & 1 & 1 & 0 \end{pmatrix} \end{array}
$$

Its face-edge incidence matrix is

$$
\boldsymbol{M}_5^{f-e} = \begin{array}{c} \\ 1 \\ 2 \\ 3 \\ 4 \\ 5 \end{array}
\begin{array}{c} \begin{array}{ccccccccc} \mathrm{a} & \mathrm{b} & \mathrm{c} & \mathrm{d} & \mathrm{e} & \mathrm{f} & \mathrm{g} & \mathrm{h} & \mathrm{i} \end{array} \\
\begin{pmatrix} 1 & 1 & 0 & 1 & 0 & 0 & 1 & 0 & 0 \\ 1 & 0 & 1 & 0 & 1 & 0 & 0 & 1 & 0 \\ 0 & 1 & 1 & 0 & 0 & 1 & 0 & 0 & 1 \\ 0 & 0 & 0 & 1 & 1 & 1 & 0 & 0 & 0 \\ 0 & 0 & 0 & 0 & 0 & 0 & 1 & 1 & 1 \end{pmatrix} \end{array}
$$

Its vertex-edge incidence matrix is

$$
\boldsymbol{M}_5^{v-e} = \begin{array}{c} \\ \mathrm{A} \\ \mathrm{B} \\ \mathrm{C} \\ \mathrm{D} \\ \mathrm{E} \\ \mathrm{F} \end{array}
\begin{array}{c} \begin{array}{ccccccccc} \mathrm{a} & \mathrm{b} & \mathrm{c} & \mathrm{d} & \mathrm{e} & \mathrm{f} & \mathrm{g} & \mathrm{h} & \mathrm{i} \end{array} \\
\begin{pmatrix} 1 & 0 & 0 & 1 & 1 & 0 & 0 & 0 & 0 \\ 0 & 1 & 0 & 1 & 0 & 1 & 0 & 0 & 0 \\ 0 & 0 & 1 & 0 & 1 & 1 & 0 & 0 & 0 \\ 1 & 0 & 0 & 0 & 0 & 0 & 1 & 1 & 0 \\ 0 & 1 & 0 & 0 & 0 & 0 & 1 & 0 & 1 \\ 0 & 0 & 1 & 0 & 0 & 0 & 0 & 1 & 1 \end{pmatrix} \end{array}
$$

Its face-vertex incidence matrix is

$$
M_5^{f-v} = \begin{array}{c} \\ 1 \\ 2 \\ 3 \\ 4 \\ 5 \end{array}
\begin{array}{c}
\begin{array}{cccccc} A & B & C & D & E & F \end{array} \\
\begin{pmatrix}
1 & 1 & 0 & 1 & 1 & 0 \\
1 & 0 & 1 & 1 & 0 & 1 \\
0 & 1 & 1 & 0 & 1 & 1 \\
1 & 1 & 1 & 0 & 0 & 0 \\
0 & 0 & 0 & 1 & 1 & 1
\end{pmatrix}
\end{array}
$$

2) The topological matrices for a 6-faced grain (00222…) generated through uphill transitions from 5-faced grain (00230…)

The grain with *p*-vector (00222…) is generated by gaining a triangular face from the 5-faced grain with *p*-vector (00230…). Fig. III-2 shows the Schlegel description of gaining a face in vertex F in the 5-faced grain. Fig. III-2(a) shows the Schlegel diagram accompanied by the generating trace, and Fig. III-2(b) is its equivalent drawing.

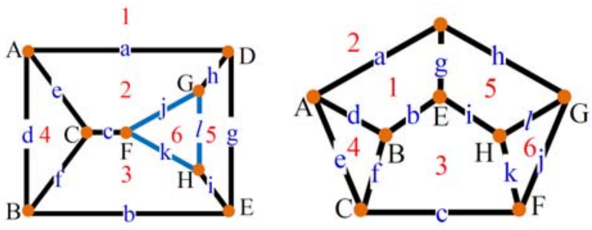

(a) (b)

Fig. III-2 the Schlegel diagram of the grain with *p*-vector of (00222…)

Its face-edge incidence matrix is

$$
M_{(00222)}^{f-e} = \begin{array}{c} \\ 1 \\ 2 \\ 3 \\ 4 \\ 5 \\ 6 \end{array}
\begin{array}{c}
\begin{array}{cccccccccccc} a & b & c & d & e & f & g & h & i & j & k & 1 \end{array} \\
\begin{pmatrix}
1 & 1 & 0 & 1 & 0 & 0 & 1 & 0 & 0 & 0 & 0 & 0 \\
1 & 0 & 1 & 0 & 1 & 0 & 0 & 1 & 0 & 1 & 0 & 0 \\
0 & 1 & 1 & 0 & 0 & 1 & 0 & 0 & 1 & 0 & 1 & 0 \\
0 & 0 & 0 & 1 & 1 & 1 & 0 & 0 & 0 & 0 & 0 & 0 \\
0 & 0 & 0 & 0 & 0 & 0 & 1 & 1 & 1 & 0 & 0 & 1 \\
0 & 0 & 0 & 0 & 0 & 0 & 0 & 0 & 0 & 1 & 1 & 1
\end{pmatrix}
\end{array}
$$

It can be seen that the portion in the red dashed rectangular is the same as M_5^{f-e}.

Its vertex-edge incidence matrix is

$$
\boldsymbol{M}^{v-e}_{(00222)} =
\begin{array}{c}
 \\ A \\ B \\ C \\ D \\ E \\ F \\ G \\ H
\end{array}
\begin{array}{c}
\begin{array}{cccccccccccc} a & b & c & d & e & f & g & h & i & j & k & l \end{array} \\
\begin{pmatrix}
1 & 0 & 0 & 1 & 1 & 0 & 0 & 0 & 0 & 0 & 0 & 0 \\
0 & 1 & 0 & 1 & 0 & 1 & 0 & 0 & 0 & 0 & 0 & 0 \\
0 & 0 & 1 & 0 & 1 & 1 & 0 & 0 & 0 & 0 & 0 & 0 \\
1 & 0 & 0 & 0 & 0 & 0 & 1 & 1 & 0 & 0 & 0 & 0 \\
0 & 1 & 0 & 0 & 0 & 0 & 1 & 0 & 1 & 0 & 0 & 0 \\
0 & 0 & 1 & 0 & 0 & 0 & 0 & 0 & 0 & 1 & 1 & 0 \\
0 & 0 & 0 & 0 & 0 & 0 & 0 & 1 & 0 & 1 & 0 & 1 \\
0 & 0 & 0 & 0 & 0 & 0 & 0 & 0 & 1 & 0 & 1 & 1
\end{pmatrix}
\end{array}
$$

It can be seen that the portion in the red dashed rectangular is the same as $\boldsymbol{M}^{v-e}_{5}$ except the row F.

3) The topological matrices for a 6-faced grain (00060…) generated through horizontal transitions from another 6-faced grain (00222…)

The grain with *p*-vector (00060…) is generated by rearrangement of faces from the grain with *p*-vector (00222…). Fig. III-3 shows the Schlegel diagram of the grain with p-vector of (00060…). The edge-switch transition is done upon the edge c in Fig. III-2.

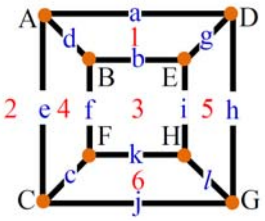

Fig. III-3 The Schlegel diagram of the grain with *p*-vector of (00060…)

Its face-edge incidence matrix is

$$
\boldsymbol{M}^{f-e}_{(00060)} =
\begin{array}{c}
 \\ 1 \\ 2 \\ 3 \\ 4 \\ 5 \\ 6
\end{array}
\begin{array}{c}
\begin{array}{cccccccccccc} a & b & c & d & e & f & g & h & i & j & k & l \end{array} \\
\begin{pmatrix}
1 & 1 & 0 & 1 & 0 & 0 & 1 & 0 & 0 & 0 & 0 & 0 \\
1 & 0 & 0 & 0 & 1 & 0 & 0 & 1 & 0 & 1 & 0 & 0 \\
0 & 1 & 0 & 0 & 0 & 1 & 0 & 0 & 1 & 0 & 1 & 0 \\
0 & 0 & 1 & 1 & 1 & 1 & 0 & 0 & 0 & 0 & 0 & 0 \\
0 & 0 & 0 & 0 & 0 & 0 & 1 & 1 & 1 & 0 & 0 & 1 \\
0 & 0 & 1 & 0 & 0 & 0 & 0 & 0 & 0 & 1 & 1 & 1
\end{pmatrix}
\end{array}
$$

Except column c, $\boldsymbol{M}^{f-e}_{(00060)}$ is the same as $\boldsymbol{M}^{f-e}_{(00222)}$.

Its vertex-edge incidence matrix is

$$
\boldsymbol{M}^{v-e}_{(00060)} = \begin{array}{c} \\ A \\ B \\ C \\ D \\ E \\ F \\ G \\ H \end{array}
\begin{array}{c} \begin{array}{cccccccccccc} a & b & c & d & e & f & g & h & i & j & k & l \end{array} \\
\begin{pmatrix}
1 & 0 & 0 & 1 & 1 & 0 & 0 & 0 & 0 & 0 & 0 & 0 \\
0 & 1 & 0 & 1 & 0 & 1 & 0 & 0 & 0 & 0 & 0 & 0 \\
0 & 0 & 1 & 0 & 1 & 0 & 0 & 0 & 0 & 1 & 0 & 0 \\
1 & 0 & 0 & 0 & 0 & 0 & 1 & 1 & 0 & 0 & 0 & 0 \\
0 & 1 & 0 & 0 & 0 & 0 & 1 & 0 & 1 & 0 & 0 & 0 \\
0 & 0 & 1 & 0 & 0 & 1 & 0 & 0 & 0 & 0 & 1 & 0 \\
0 & 0 & 0 & 0 & 0 & 0 & 0 & 1 & 0 & 1 & 0 & 1 \\
0 & 0 & 0 & 0 & 0 & 0 & 0 & 0 & 1 & 0 & 1 & 1
\end{pmatrix} \end{array}
$$

Except the portions in blue circles, $\boldsymbol{M}^{v-e}_{(00060)}$ is the same as $\boldsymbol{M}^{v-e}_{(00222)}$.

IV. Proofs for the viewpoint of “one of *f*-faced forms can be transformed to all of the other forms with *f* faces by the horizontal edge switch transitions”

The matrix description of grain form evolution in grain growth is able to generate all of the possible grain forms, in that any one of *f*-faced forms can be transformed to all of the other forms with *f* faces by the horizontal edge switch transitions, i.e., rearrangement of faces. Patterson has mentioned this rationale briefly in Ref. [13]. In order to illustrate it more clearly, we give a detail proof as follows.

According to the topology event of rearrangement of grain faces, a polygonal face will lose an edge if edge-switching transition is conducted on any one of its edges (see Fig. IV-1). And, a polygonal face will gain an edge if edge-switching transition is conducted on any one of its connected edges (see Fig. IV-2). Thus, the faces of a grain form can be rearranged by edge-switching transition.

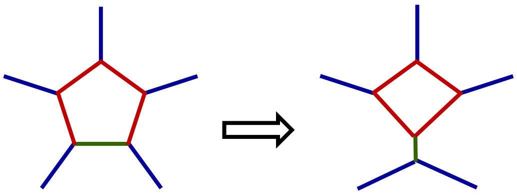

Fig. IV-1 A face loses an edge after one of its edges doing edge-switching transition. The face is closed by red and green edges before the transition, and it is closed only by red edges after the transition. The green edge denotes the switching edge.

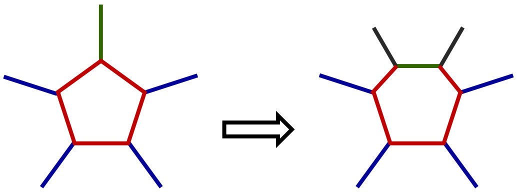

Fig. IV-2 A face gains an edge after one of its connected edges doing edge-switching transition. The face is closed by red edges before the transition, and it is closed by red and green edges after the transition. The green edge denotes the switching edge.

Euler’s Law relates the faces, edges, and vertices of a polyhedron as (n-q+p=2) [17]. Under the restrictions of surface tension in three-dimensional cellular structures, three boundary interfaces meet at an

edge and four edges meet at a vertex. For an individual grain, each edge has two vertices and each vertex radiates three edges, i.e., $2q=3p$ [3]. We can get $p=2(n-2)$ and $q=3(n-2)$. Thus, for a certain face class n, the edges q and vertices p are all invariable during the rearrangement of grain faces. This ensures that the number of grain forms in a certain finite face class is definite. Consequently, one grain form could be evolved to the other one in a finite step because the face number of each grain forms is finite.

As an example, Fig.IV-3 shows the transition process of two forms of 7-faced grains by one step of edge-switching transition. It is made more clearly in Fig.IV-4, showing the grain form evolution in 7-faced class by one-step edge-switching transition. The path of the transition between any two 7-faced grain forms can be seen by the rearrangement of faces. The grain forms and their relationships in Fig.IV-4 can also be regard as a graph in graph theory. Actually, the algorithm of generating all of the possible grain forms is a breadth first search (BFS) for graph traversal according to the illustration by Fig.IV-4.

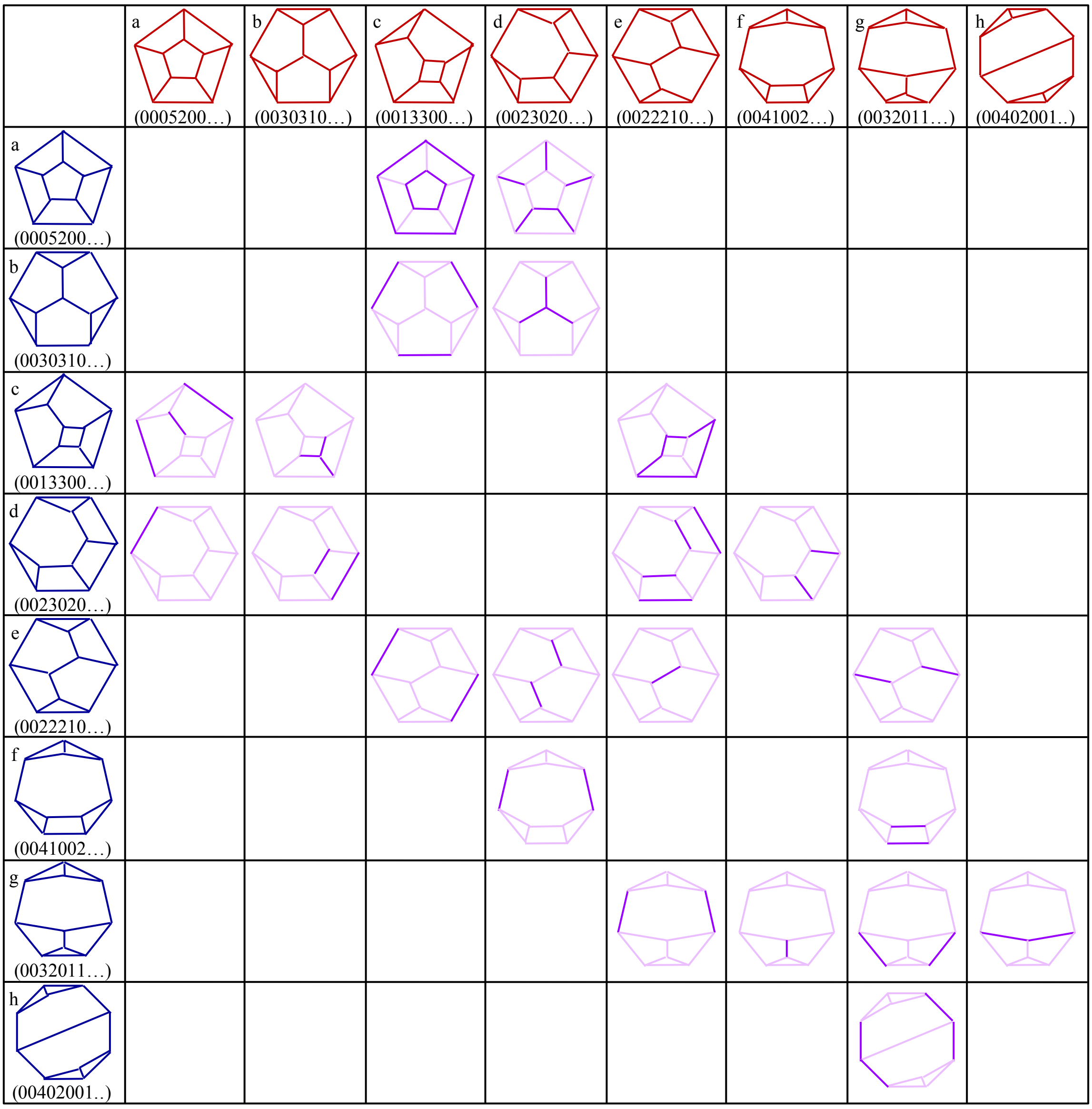

Fig.IV-3 Transitions between two forms of 7-faced grains by one step of edge-switching transition. The dark purple lines denote the optional switching edges in the transition of the blue forms to the red ones.

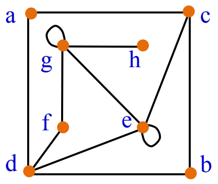

Fig.IV-4 Plot of the grain form evolution in 7 face class by one-step edge-switching transition. The path of the transition between any two 7-faced grain forms can be seen.

V. Schlegel diagram, *p*-vector, and algebraic connectivity (λ_2) of 8- and 9-faced grains

8-faced grain

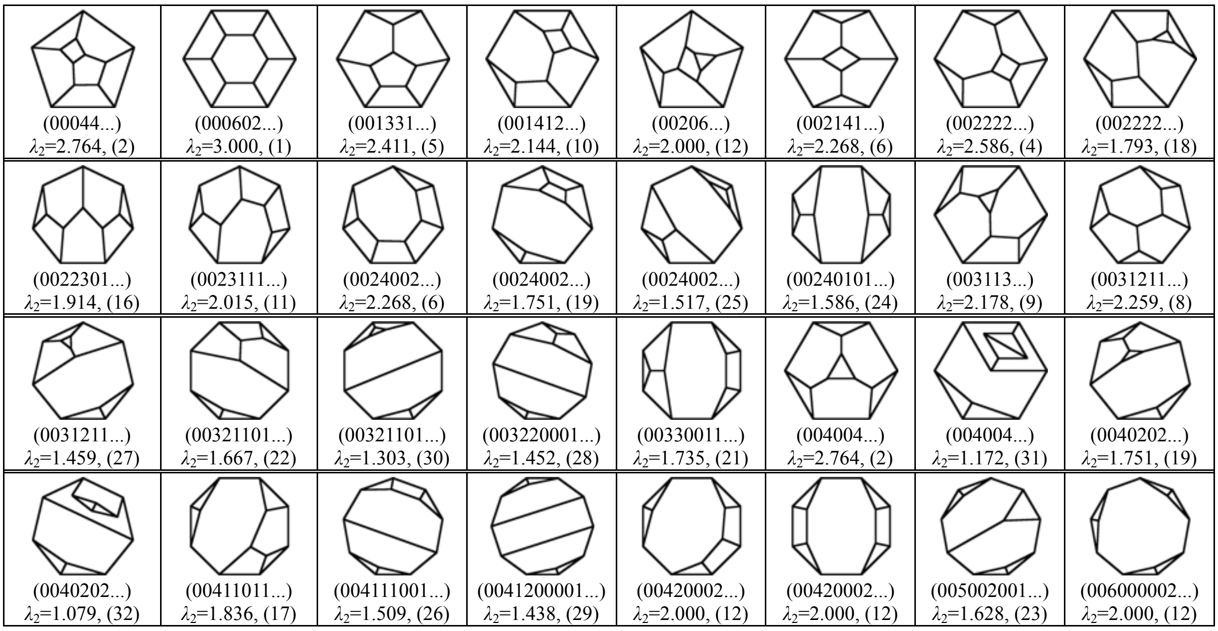

(00044...) λ_2=2.764, (2)	(000602...) λ_2=3.000, (1)	(001331...) λ_2=2.411, (5)	(001412...) λ_2=2.144, (10)	(00206...) λ_2=2.000, (12)	(002141...) λ_2=2.268, (6)	(002222...) λ_2=2.586, (4)	(002222...) λ_2=1.793, (18)
(0022301...) λ_2=1.914, (16)	(0023111...) λ_2=2.015, (11)	(0024002...) λ_2=2.268, (6)	(0024002...) λ_2=1.751, (19)	(0024002...) λ_2=1.517, (25)	(00240101...) λ_2=1.586, (24)	(003113...) λ_2=2.178, (9)	(0031211...) λ_2=2.259, (8)
(0031211...) λ_2=1.459, (27)	(00321101...) λ_2=1.667, (22)	(00321101...) λ_2=1.303, (30)	(003220001...) λ_2=1.452, (28)	(00330011...) λ_2=1.735, (21)	(004004...) λ_2=2.764, (2)	(004004...) λ_2=1.172, (31)	(0040202...) λ_2=1.751, (19)
(0040202...) λ_2=1.079, (32)	(00411011...) λ_2=1.836, (17)	(004111001...) λ_2=1.509, (26)	(0041200001...) λ_2=1.438, (29)	(00420002...) λ_2=2.000, (12)	(00420002...) λ_2=2.000, (12)	(005002001...) λ_2=1.628, (23)	(006000002...) λ_2=2.000, (12)

9-faced grain

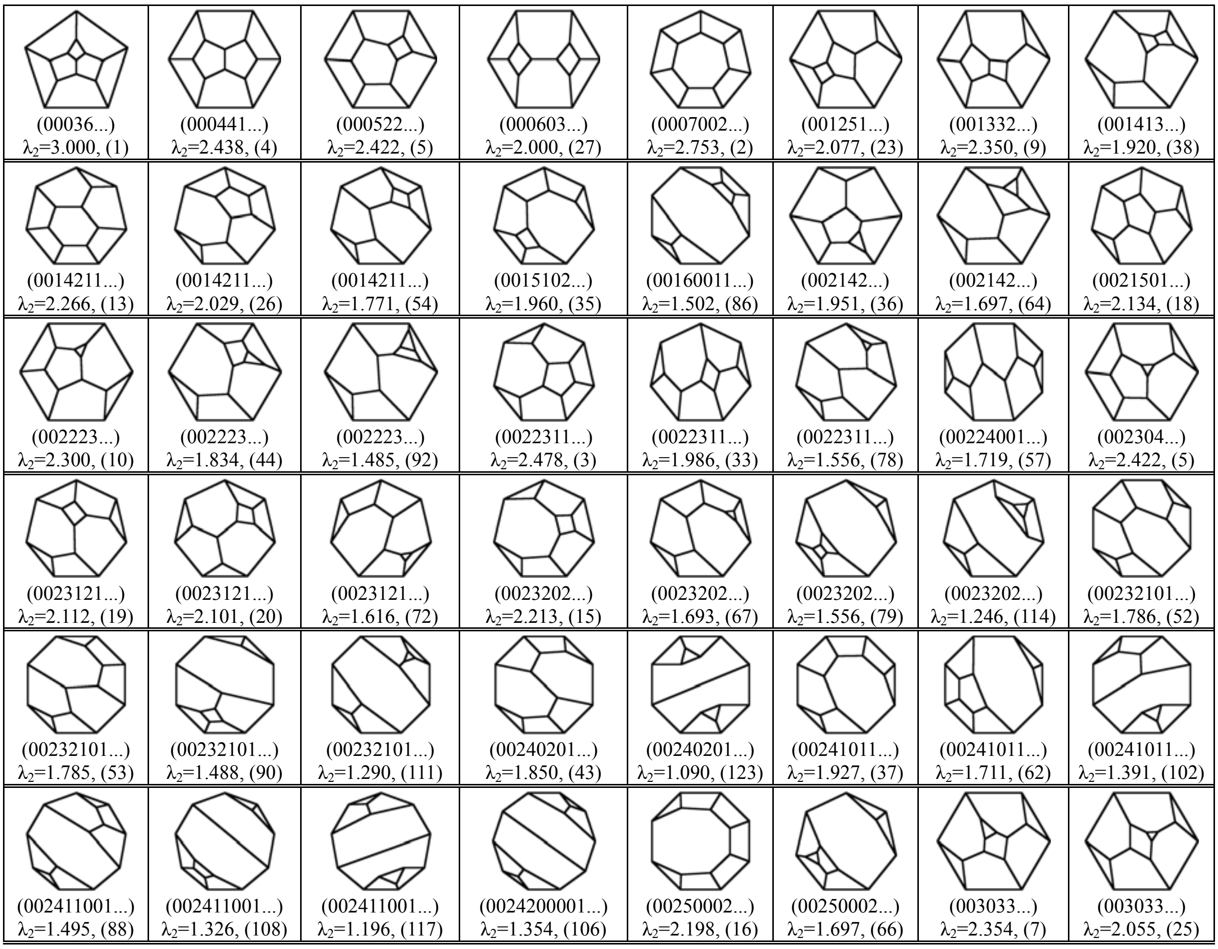

(00036...) λ_2=3.000, (1)	(000441...) λ_2=2.438, (4)	(000522...) λ_2=2.422, (5)	(000603...) λ_2=2.000, (27)	(0007002...) λ_2=2.753, (2)	(001251...) λ_2=2.077, (23)	(001332...) λ_2=2.350, (9)	(001413...) λ_2=1.920, (38)
(0014211...) λ_2=2.266, (13)	(0014211...) λ_2=2.029, (26)	(0014211...) λ_2=1.771, (54)	(0015102...) λ_2=1.960, (35)	(00160011...) λ_2=1.502, (86)	(002142...) λ_2=1.951, (36)	(002142...) λ_2=1.697, (64)	(0021501...) λ_2=2.134, (18)
(002223...) λ_2=2.300, (10)	(002223...) λ_2=1.834, (44)	(002223...) λ_2=1.485, (92)	(0022311...) λ_2=2.478, (3)	(0022311...) λ_2=1.986, (33)	(0022311...) λ_2=1.556, (78)	(00224001...) λ_2=1.719, (57)	(002304...) λ_2=2.422, (5)
(0023121...) λ_2=2.112, (19)	(0023121...) λ_2=2.101, (20)	(0023121...) λ_2=1.616, (72)	(0023202...) λ_2=2.213, (15)	(0023202...) λ_2=1.693, (67)	(0023202...) λ_2=1.556, (79)	(0023202...) λ_2=1.246, (114)	(00232101...) λ_2=1.786, (52)
(00232101...) λ_2=1.785, (53)	(00232101...) λ_2=1.488, (90)	(00232101...) λ_2=1.290, (111)	(00240201...) λ_2=1.850, (43)	(00240201...) λ_2=1.090, (123)	(00241011...) λ_2=1.927, (37)	(00241011...) λ_2=1.711, (62)	(00241011...) λ_2=1.391, (102)
(002411001...) λ_2=1.495, (88)	(002411001...) λ_2=1.326, (108)	(002411001...) λ_2=1.196, (117)	(0024200001...) λ_2=1.354, (106)	(00250002...) λ_2=2.198, (16)	(00250002...) λ_2=1.697, (66)	(003033...) λ_2=2.354, (7)	(003033...) λ_2=2.055, (25)

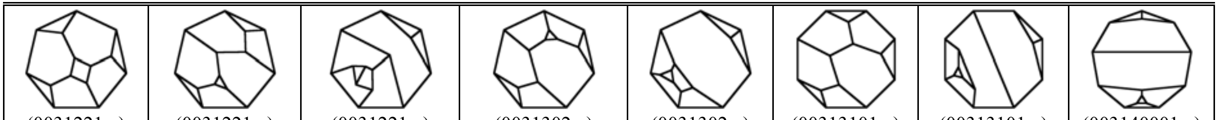

(0031221...)<br>λ_2=2.354, (7)	(0031221...)<br>λ_2=1.787, (51)	(0031221...)<br>λ_2=1.261, (112)	(0031302...)<br>λ_2=1.823, (46)	(0031302...)<br>λ_2=1.484, (93)	(00313101...)<br>λ_2=1.912, (39)	(00313101...)<br>λ_2=1.101, (122)	(003140001...)<br>λ_2=1.208, (116)

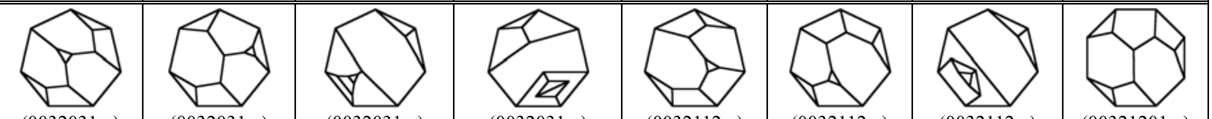

(0032031...)<br>λ_2=2.000, (27)	(0032031...)<br>λ_2=1.871, (41)	(0032031...)<br>λ_2=1.303, (110)	(0032031...)<br>λ_2=1.028, (125)	(0032112...)<br>λ_2=2.086, (21)	(0032112...)<br>λ_2=1.976, (34)	(0032112...)<br>λ_2=0.923, (130)	(00321201...)<br>λ_2=2.060, (24)

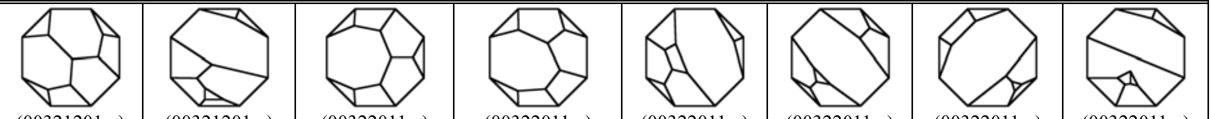

(00321201...)<br>λ_2=2.000, (27)	(00321201...)<br>λ_2=1.408, (97)	(00322011...)<br>λ_2=2.289, (11)	(00322011...)<br>λ_2=2.080, (22)	(00322011...)<br>λ_2=1.715, (58)	(00322011...)<br>λ_2=1.501, (87)	(00322011...)<br>λ_2=1.383, (103)	(00322011...)<br>λ_2=1.174, (119)

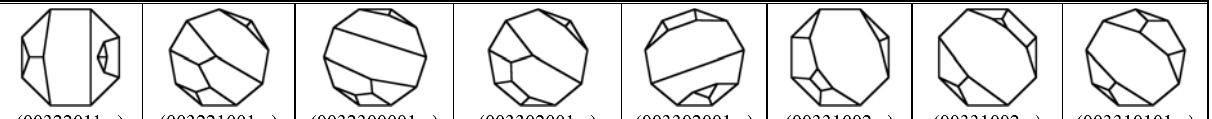

(00322011...)<br>λ_2=0.979, (127)	(003221001...)<br>λ_2=1.580, (77)	(0032300001...)<br>λ_2=1.406, (98)	(003302001...)<br>λ_2=1.614, (73)	(003302001...)<br>λ_2=1.236, (115)	(00331002...)<br>λ_2=1.802, (48)	(00331002...)<br>λ_2=1.554, (80)	(003310101...)<br>λ_2=1.611, (75)

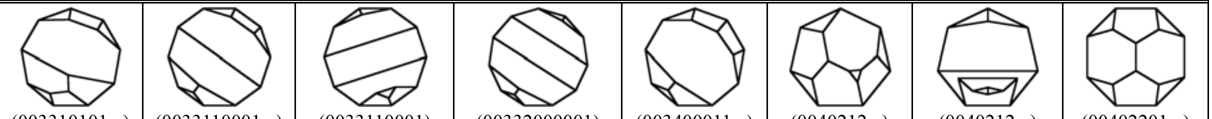

(003310101...)<br>λ_2=1.614, (74)	(0033110001...)<br>λ_2=1.401, (99)	(0033110001)<br>λ_2=1.196, (118)	(00332000001)<br>λ_2=1.366, (105)	(003400011...)<br>λ_2=1.709, (63)	(0040212...)<br>λ_2=2.192, (17)	(0040212...)<br>λ_2=0.819, (131)	(00402201...)<br>λ_2=2.268, (12)

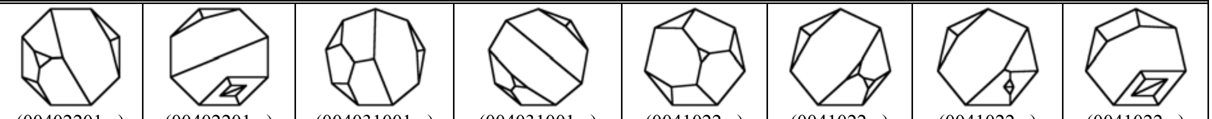

(00402201...)<br>λ_2=1.488, (89)	(00402201...)<br>λ_2=0.960, (129)	(004031001...)<br>λ_2=1.632, (70)	(004031001...)<br>λ_2=1.321, (109)	(0041022...)<br>λ_2=2.238, (14)	(0041022...)<br>λ_2=1.522, (83)	(0041022...)<br>λ_2=1.150, (120)	(0041022...)<br>λ_2=1.090, (123)

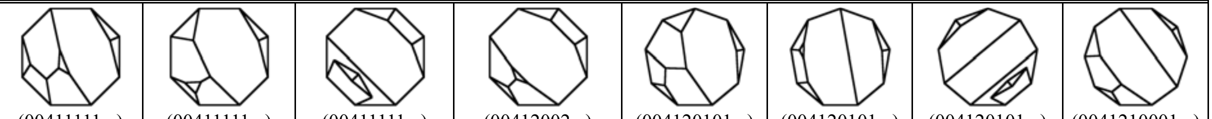

(00411111...)<br>λ_2=1.713, (61)	(00411111...)<br>λ_2=1.532, (82)	(00411111...)<br>λ_2=1.006, (126)	(00412002...)<br>λ_2=1.697, (64)	(004120101...)<br>λ_2=1.714, (59)	(004120101...)<br>λ_2=1.335, (107)	(004120101...)<br>λ_2=0.971, (128)	(0041210001...)<br>λ_2=1.453, (96)

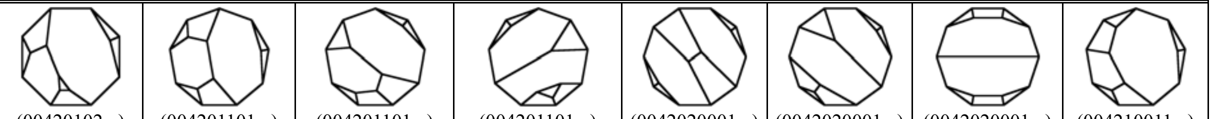

(00420102...)<br>λ_2=1.823, (45)	(004201101...)<br>λ_2=1.788, (50)	(004201101...)<br>λ_2=1.735, (56)	(004201101...)<br>λ_2=1.369, (104)	(0042020001...)<br>λ_2=1.551, (81)	(0042020001...)<br>λ_2=1.502, (85)	(0042020001...)<br>λ_2=1.459, (94)	(004210011...)<br>λ_2=1.854, (42)

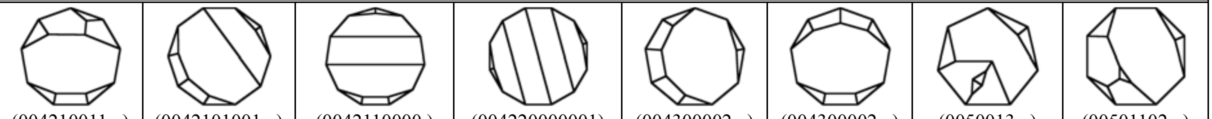

(004210011...)<br>λ_2=1.802, (49)	(0042101001...)<br>λ_2=1.486, (91)	(0042110000.)<br>λ_2=1.400, (100)	(004220000001)<br>λ_2=1.398, (101)	(004300002...)<br>λ_2=2.000, (27)	(004300002...)<br>λ_2=2.000, (27)	(0050013...)<br>λ_2=1.248, (113)	(00501102...)<br>λ_2=1.804, (47)

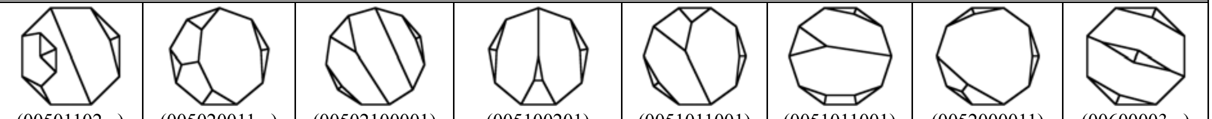

(00501102...)<br>λ_2=1.146, (121)	(005020011...)<br>λ_2=1.881, (40)	(00502100001)<br>λ_2=1.454, (95)	(005100201)<br>λ_2=1.683, (68)	(0051011001)<br>λ_2=1.629, (71)	(0051011001)<br>λ_2=1.586, (76)	(0052000011)<br>λ_2=1.713, (60)	(00600003...)<br>λ_2=1.757, (55)

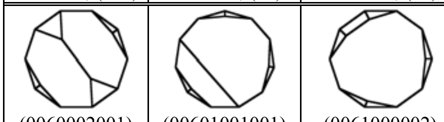

(0060002001)<br>λ_2=1.683, (68)	(00601001001)<br>λ_2=1.510, (84)	(0061000002)<br>λ_2=2.000, (27)					
